# Porcine Corneal Tissue and Xenozoonotic Risks: A Review of the Current Evidence

**DOI:** 10.1111/xen.70068

**Published:** 2025-11-07

**Authors:** Rodrigo Moreira, Heloisa Nascimento, Thaís Maria da Mata Martins, Gabriel Barbieri, Pedro Pires, Lucimeire N. Carvalho, Larissa R. Rosa, Augusto Almeida, Carmen Luz Pessuti, Henrique Ferrer, José Álvaro Pereira Gomes, Ernesto Goulart, Silvano Raia, Rubens Belfort

**Affiliations:** ^1^ Vision Institute (IPEPO) São Paulo Brazil; ^2^ Federal University of São Paulo (UNIFESP) São Paulo Brazil; ^3^ Federal University of Viçosa (UFV) Viçosa Brazil; ^4^ University of São Paulo (USP) São Paulo Brazil

**Keywords:** corneal transplant, xenotransplantation, xenozoonosis

## Abstract

Corneal opacities affect millions worldwide, with corneal transplantation as the primary treatment. However, donor shortages remain a challenge, leaving thousands waiting for transplants. Xenotransplantation using porcine corneas has emerged as a promising alternative due to anatomical and physiological similarities with human corneas. Advances in CRISPR/Cas technology enable genetic modifications to address immune rejection and zoonotic risks. A key concern is xenozoonosis, the transmission of infectious agents from pigs to humans. Pathogens such as porcine endogenous retroviruses (PERVs), porcine cytomegalovirus (PCMV), and bacteria pose potential risks. While PERVs can infect human cells in vitro, no transmission has been documented in vivo. Regulatory bodies, including the WHO and IXA, have established guidelines for monitoring and clinical trials. The first human corneal xenotransplantation trials in South Korea and China are underway. Enhanced biosecurity measures in tissue banks have reduced microbial contamination, improving safety. Corneal xenotransplantation presents lower risks compared to solid organ xenotransplants. With ongoing research, stringent regulations, and improved pathogen‐free animal models, this technique could become a viable clinical option. Future human trials will provide crucial insights into its long‐term safety and effectiveness.

**Abbreviations**
CRISPR/Cas: clustered regularly interspaced short palindromic repeats associated with Cas nucleases
EBBA: European Eye Bank Association
IXA: International Xenotransplantation Association
NHP: non‐human primates
PARV: parvovirus
PCMV: porcine cytomegalovirus
PCV: porcine circovirus
PERV: porcine endogenous retroviruses
PLHV: porcine lymphotropic herpes virus
WHO: World Health Organization

## 1 Introduction

Corneal opacities affect approximately 2.9 million people with moderate or severe visual impairment [1]. The primary treatment for these conditions continues to be corneal transplantation [2]. In Brazil, despite significant growth in the number of transplants performed, it is estimated that around 24 000 patients are waiting for the procedure [3, 4].

Recently, xenotransplantation, a medical procedure in which living tissues or organs from a non‐human animal source are transplanted into a human recipient, has emerged as a potential alternative to allotransplantation [5, 6]. This is due to the cornea's well‐documented immune privilege [7] and the advent of Clustered Regularly Interspaced Short Palindromic Repeats associated with Cas nucleases (CRISPR/Cas) technology, which has enabled genetic modifications to overcome challenges such as immune rejection, species incompatibility, and xenozoonoses [8, 9, 10].

Additionally, it is already known that the ideal animal model for this procedure is the pig, as the corneas of these animals exhibit anatomical, physiological, and optical characteristics similar to those of humans [11].

However, some ethical and safety concerns still surround this field of study [12]. One such concern is the possibility of xenozoonosis transmission from the donor to the recipient, which could be a limiting factor in the success of xenotransplantation [5].

Significant challenges remain due to the lack of data on swine microbiology and the absence of standardized methods to track and monitor potential pathogens. Thus, our study aims to investigate the literature regarding the infectious agents in conventional and genetically modified porcine corneas.

## 2 Ethical and Safety Issues

Although xenotransplantation has the potential to revolutionize the field of allotransplantation, it brings forth ethical concerns that need to be addressed [13, 14]. The use of animals in scientific research has been a topic of extensive debate, with various arguments presented in both public and academic circles [15]. At present, the use of non‐human animal‐derived products, such as heart valves, insulin, and tissues like skin grafts for burn victims, plays a vital role in justifying these practices [15].

Furthermore, given the uncertainty about the absolute risk of xenotransplants and xenozoonotic infections, the implementation of common guidelines to regulate experiments has become essential [16]. In 2004, the World Health Organization (WHO) recommended that member states harmonize their practices, allowing xenotransplantation only when effective control and regulatory surveillance mechanisms were established [17]. In addition, in cases of successful xenotransplantation, recipients and their contacts are likely to undergo lifelong surveillance to detect and treat any unexpected adverse effects, even if the xenotransplanted donor organ is removed [5].

Subsequently, the International Xenotransplantation Association (IXA) developed guidelines addressing both ethical aspects and the preclinical requirements necessary to ensure proper oversight by human and animal authorities [18]. Currently, countries such as the United States [19], China [20], South Korea [21], Japan [22], the United Kingdom [23], and European Union members [24, 25] have established their own regulations or guidelines for the clinical use of xenoproducts.

All the regulations of the countries listed above operate based on certain key pillars for the approval of clinical trials involving xenoproducts. These regulations are based on principles such as the potential risk of infection for contact populations, the breeding of donor animals, as well as testing of the tissue to be used, the risk‐benefit analysis for the recipient, and the follow‐up of the patient from the preoperative to the postoperative period [19, 20, 21, 22, 23, 24, 25].

### 2.1 Current Regulation

Regarding corneal xenotransplantation, it was only in 2014 that IXA proposed specific recommendations for clinical trials involving pig corneas, establishing strategies that include monitoring the expression of porcine endogenous retrovirus (PERV) in pigs and porcine grafts, as well as long‐term post‐transplant follow‐up of the recipient with blood sample analysis [26]. It was also established that cases of zoonotic infections must be mandatorily reported by the national regulatory body after confirmation by laboratory tests [26].

In 2019, Korean researchers developed the first clinical protocol for corneal xenotransplantation [27]. Assuming the risk of xenozoonotic transmission combined with intense systemic immunosuppression, continuous monitoring measures will be adopted, extending to the patient's spouses or sexual partners. Periodic samples will be collected to assess immune responses, changes in anti‐Gal antibodies, the presence of PERV material in the blood, and C3a levels in the aqueous humor [27].

Recently, corneal transplants performed in Rio de Janeiro [28], Brazil, in which the donors inadvertently had HIV, have sparked discussions about the safety of eye and tissue banks. Although no corneal tissue was affected, most likely due to the immunophysiological privilege of the cornea, the issue raises concerns about the challenges of oversight and the need for regulations in the field of allotransplants as well.

### 2.2 Psychological and Social Perception Challenges

After a transplant, patients may experience various psychological and social perception challenges, which are well‐documented in the medical literature. This stress can be intensified by episodes of graft rejection or infections, increasing levels of anxiety and depression [29]. Additionally, how patients perceive the graft as part of their own body or as a foreign entity can affect their mental well‐being. While most patients successfully integrate the graft into their body image, some may experience emotional stress related to the graft or the donor [30]. Medical literature highlights the effectiveness of psychosocial interventions in improving psychological symptoms and treatment adherence among transplant recipients. For instance, a systematic review and meta‐analysis showed that such interventions effectively reduce depression and anxiety in kidney and heart transplant recipients, especially when provided postoperatively [31]. Psychological support programs, including pre‐transplant consultations with psychologists, social workers, and other professionals, are recommended to prepare patients and their families for the transplant process, helping to mitigate negative experiences and enhance the overall transplant experience [32].

Nonetheless, a prospective multicenter study found that corneal transplantation has a positive impact on mental health outcomes over time, with significant improvements in symptoms of depression and anxiety up to 24 months after the procedure. However, certain factors were identified as predictors of poorer mental health, including comorbidities, ocular complaints (such as dry eye), and passive coping styles [33].

## 3 Potential Infectious Agents

Xenozoonosis is the term used to describe the infection of humans by pathogens originating from animals used in xenotransplantation, such as in corneal transplants of porcine origin [34, 35]. Currently, techniques have been developed to isolate infectious agents from animal embryos and produce disease‐free animals, known as “pathogen‐free pigs” [36].

The full spectrum of pathogens that can infect the human host or cause disease in transplanted porcine organs/cells has not yet been fully defined [1]. However, Otabi et al. in 2023 developed a list of potential pathogens that could potentially infect donor pigs [37]. In this way, the following will address the infectious agents in genetically modified and conventional pigs that could pose risks for xenozoonosis, especially in corneal grafts.

### 3.1 Porcine Endogenous Retroviruses

It represents a group composed of three retrovirus subtypes (PERV‐A, PERV‐B, and PERV‐C) integrated into the pig genome [38]. Studies have demonstrated that PERVs have the ability to infect human cells in vitro, including the cornea [39]. However, no cases of endogenous retrovirus transmission to non‐human primates (NHP) have been observed in studies involving the transplantation of porcine cells, tissues, or organs [40, 41]. Studies on corneas have also shown no evidence of transmission [13]. This evidence demonstrated that some NHP species exhibit a lack of susceptibility to PERVs, which invalidates them as suitable models to demonstrate infection [41]. Even with the advances in bioengineering related to CRISPR/Cas, PERVs have not yet been completely eliminated from genetically modified pigs [14]. Nevertheless, Li et al. suggest that using the Wuzhishan miniature pig as a donor may be advantageous since it lacks PERV‐C and, therefore, does not produce potentially infectious A/C recombinants [38].

### 3.2 Porcine Cytomegalovirus

Also known as Suid herpesvirus 2, it is an enveloped virus with a double‐stranded linear DNA genome [42]. Infections can cause consumptive coagulopathy and early renal graft loss in pig‐to‐non‐human primate transplants, and it appears to be resistant to ganciclovir treatment [35]. It has been found that infection of donor pigs with porcine cytomegalovirus (PCMV) significantly reduces graft survival in pig‐to‐NHP xenotransplantation, decreasing survival rates by two to three times [43]. Recently, it was discovered that PCMV can be eliminated from herds through early weaning of piglets and artificial feeding [44, 45].

### 3.3 Porcine Lymphotropic Herpes Virus

It is difficult to eliminate from swine herds but has not been associated with diseases in xenotransplants with immunosuppression [46]. However, porcine lymphotropic herpes virus (PLHV)‐1 DNA was detected in peripheral blood mononuclear cells and plasma of recipient baboons after pig solid organ transplantation [40]. The development of an ELISA‐based serological test for PLHV has added an important tool for screening donor pigs [47].

### 3.4 Parvovirus

Researchers identified a new human parvovirus, termed PARV‐4, in the plasma of a patient exhibiting symptoms similar to acute HIV infection [48]. PARV‐4 has also been detected in blood donors and blood‐derived products, and evidence shows it can be transmitted through blood transfusion [49]. Its detection in human plasma points to a viremic phase, which could enable the virus to spread to various organs. A comparable parvovirus has been found in pigs [50], and considering the findings discussed above, there is a potential risk of its transmission via xenotransplantation [51].

### 3.5 Porcine Circovirus

Porcine circoviruses (PCVs) are small, single‐stranded DNA viruses commonly found in pigs and include several species such as PCV1, PCV2, PCV3, and PCV4 [51]. PCV3, a more recently identified species, has been found in both commercial pigs and pigs bred for xenotransplantation [52]. Studies have demonstrated the transmission of PCV3 from pigs to baboons through cardiac transplants, indicating the virus's ability to cross species barriers [52]. Although the pathogenicity of PCV3 in pigs was initially controversial, inoculation studies have confirmed its capacity to cause disease [53].

### 3.6 Alphaherpesviruses

Compared to the herpes virus in humans, this agent appears to interact with the nasal mucosa of pigs, its primary site of replication, by activating cellular serine proteases that create openings in the basement membrane, allowing the virus to spread rapidly in the lamina propria and submucosa [54]. Subsequently, alphaherpesviruses spread along the axons to the neuronal cell body of sensory neurons [55].

### 3.7 Suid Herpesvirus 1

Aujeszky's disease, also known as pseudorabies, belongs to the Alphaherpesvirinae subfamily [56]. It is characterized by nervous and respiratory clinical signs, piglet mortality, and severe reproductive disorders in pregnant sows. The main reproductive clinical signs observed include fetal resorption, abortions, malformations, infertility, and the birth of weak piglets [56].

### 3.8 Staphylococcus Aureus

Pigs have been identified as a source of methicillin‐resistant *S. aureus*, reflecting the range of diseases that *S. aureus* can be involved in [57, 58]. Furthermore, other gram‐positive bacteria were found in corneal cultures of genetically modified pigs [59].

### 3.9 Clostridium Difficile

A study looked into the presence of *C. difficile* in pigs used for skin grafts in xenotransplantation [60]. The bacteria were found in the feces of healthy pigs used to treat burn patients, although they were not found on the skin itself [60]. Since some pig strains of *C. difficile* are very similar to those found in humans, there is a high risk of cross‐species infection [61]. These findings show that testing donor pigs for *C. difficile* is important for safety [61].

### 3.10 Haemophilus Parasuis

It is a gram‐negative coccobacillus belonging to the family Pasteurellaceae and the causative agent of Glässer's Disease, which is characterized by the deposition of fibrinous to fibrinosuppurative material in joints, serous membranes, and meninges. Histopathological findings suggest the possibility of the coccobacillus crossing hematogenous barriers in different organs such as the brain, eye, and thymus. Additionally, ocular lesions never before described for Glässer's Disease were observed in several animals and were characterized by endophthalmitis and perineuritis of the optic nerve [62].

### 3.11 Chlamydiae

They are a group of obligate intracellular pathogens that cause diseases in a wide range of host species. *Chlamydia suis*, belonging to the family Chlamydiaceae, is naturally found in pigs and is genetically close to *Chlamydia trachomatis*, an important human ocular pathogen. Chahota et al. detected an unexpectedly high genetic diversity of *C. suis* among pigs tested with conjunctivitis. These findings and the present results indicate the varied pathogenic potential of this bacterium, which appears to survive well and potentially induce pathologies in the ocular anatomical niche [63].

### 3.12 Toxoplasma Gondii

It is a protozoan parasite that normally causes a subclinical infection in most animal species; however, a primary infection during pregnancy can cause fetal pathologies, as well as abortions in humans and some animal species [64]. Ocular toxoplasmosis is another concern with *T. gondii* infection, and this may occur following congenital and acquired transmission [64]. Garcia et al. conducted a study where pigs were infected with *T. gondii* tachyzoites, bradyzoites, and oocysts [65]. They demonstrated that the infection in pigs depends on the breed and age of the animals, the life cycle stage of the parasite responsible for the infection, the route of infection, and the infectious dose of the parasites. However, in this study, no pigs were diagnosed with the disease through ELISA and PCR methods [65].

### 3.13 Acanthamoeba

It represents a family of parasites that potentially cause ocular infections. He et al. introduced infected contact lenses in micropigs. Three distinct stages of the disease became evident and were categorized as acute, condensed infiltrate, and resolution stages. Additionally, cysts were identified deep within the stroma of histological specimens collected during the resolution stages. The dense, white ring‐like infiltrates, stromal edema, keratic precipitates, and the chronic nature of the infections were similar to those observed in human *Acanthamoeba keratitis*, showing a strong correlation between the clinical and histopathological features of contact lens‐induced *A. keratitis* in pigs and humans [66].

Although all the pathogens listed above present a potential risk of xenozoonosis, special attention should be given to viruses, especially PERVs, PCMV, and PLHV. Although none of them has been associated with ocular infection in xenotransplantation, these pathogens are the most studied due to their virtual risk of xenozoonosis.

### 3.14 Detection Methods and Follow‐Up in Xenotransplantation

To prevent infections caused by the potential pathogens listed above, it is necessary to use highly sensitive methods to maintain pathogen‐free xenodonor herds and to screen xenografts prior to transplantation [37]. This process is challenging, as confirming the presence or absence of certain pathogens, especially latent viruses, requires a validated arsenal of direct and indirect tests [53]. In a study conducted in 2020 by Wang et al., it was demonstrated that the detection of PCMV using a one‐tube nested real‐time PCR method showed high sensitivity and a shorter analysis time compared to other detection methods [43].

In xenotransplant recipients, the risks of infection and rejection will require lifelong monitoring [5]. Monitoring schemes to detect known pathogens are being developed, especially for those with a higher risk of association with xenozoonosis (PERV and herpesviruses) [44], even in the absence of clinical evidence of infection [13]. These schemes are being based on the use of new molecular techniques such as broad‐range hybridization probes or PCR primers, molecular differential display, and microarray technologies [44].

Given the possibility that unexpected clinical symptoms may result from xenogeneic infection, prepared responses are required that would not differ significantly from the approach taken for allotransplant recipients [5]. That is, routine bacterial, fungal, and viral cultures performed on cells of both human and porcine origin before the initiation of antimicrobial therapy; PCR for PERV and herpesviruses using both serum and leukocytes; and coculture of peripheral blood leukocytes with human and donor cell lines [44]. This is followed by empirical antimicrobial therapy and hospital admission with isolation from other patients until the nature of the process is better defined [44].

### 3.15 Infectious Agents in Corneal Allogeneic Transplantation

In the context of allogeneic corneal transplantation, studies indicate that a significant proportion of post‐transplant infections may be attributed to donor‐to‐recipient transmission [67]. The most commonly associated infectious agents include primarily gram‐negative bacteria, fungi, and, to a lesser extent, viruses. According to the medical literature, bacterial infections are predominant, with a high incidence of gram‐negative organisms such as Pseudomonas aeruginosa and other multidrug‐resistant bacteria [68, 69]. Fungal infections, particularly those caused by Candida species, represent a significant concern, especially due to the lack of antifungal agents in corneal preservation media [70, 71]. Moreover, a notable association has been documented between the storage of corneas in hypothermic media and a higher incidence of fungal infections [72, 73].

### 3.16 Advancing Safety Measures and Protocols for Corneal Tissue in Xenotransplantation

In 2016, Kim et al. demonstrated that, in the culture of non‐genetically modified pig corneas, 30% tested positive for bacteria, all gram‐positive: *Aerococcus viridans*, *Leuconostoc pseudomesenteroides*, and *Staphylococcus hominis*, using the standard operating procedure recommended by the European Eye Bank Association (EEBA), a technical guideline for ocular tissues [59].

However, when using a modified EEBA protocol, with the addition of antiseptic procedures such as immersion in a 5% betadine solution for 1 min and irrigation with 10% cefazolin and 2% gentamicin solutions, no microorganisms were detected. Thus, it was concluded that it is possible to obtain xenocorneal grafts free of bacteria, fungi, and protozoa using the modified EEBA protocol [59].

#### 3.16.1 The Role of CRISPR/Cas Technology

This technological breakthrough in genetic engineering made it possible to perform multiple genetic modifications in a single animal to address problems like immune rejection, species incompatibility, and the risk of xenozoonotic infections [10]. The most extensive gene edits using CRISPR/Cas9 for xenotransplantation were achieved in 2020, when pigs were created with four pig genes turned off (GGTA1, CMAH, β4GalNT2, and β2M) and five human genes inserted: CD46, CD55, CD59, HO1, and A20. More recently, multi‐transgenic pigs have been developed with inactive pig genes and expression of new human genes, as well as the ability to deactivate up to 25 copies of PERV in the same animals [74].

Research using this technique in corneal xenotransplantation is also underway. Choi et al. achieved pig‐to‐monkey corneal graft survival of up to 933 days with anti‐CD40 immunosuppression [75]. Similarly, Kim et al. reported graft survival of 511 and 470 days using anti‐CD40 and anti‐CD20 antibodies, respectively [76].

## 4 Corneal Xenozoonosis: What We Know

In 2017, Hyuk et al. conducted a study aimed at demonstrating the long‐term safety of PERV transmission following corneal transplants from pigs to NHP. This study confirmed that keratinocytes from non‐genetically modified pigs exhibited genetic sequences related to PERVs, which implies the presence of PERVs‐expressing cells in porcine corneal tissues [13]. However, when assessing the infectivity of PERVs in keratinocytes of porcine corneas xenotransplanted into rhesus monkeys, no evidence of PERVs transmission was found in vitro, nor was there any infection of this agent through blood and plasma samples after 1176 days of follow‐up [13]. Therefore, it was concluded that there is no evidence supporting any risk of PERVs transmission from porcine corneal tissues to NHP recipients, despite the presence of PERVs expression in porcine corneal cells [13].

Although no studies have demonstrated the occurrence of xenozoonosis in humans through corneal xenotransplantation, the first clinical trials of genetically modified pig xenotransplantation in humans are being conducted in South Korea and China, promising to further elucidate this field [77, 78].

## 5 Conclusion

Although there is a theoretical risk of viral xenozoonosis, especially PERVs, through corneal xenotransplantation [13], there has been no documentation of infection in humans or NHPs to date [77]. In addition, infection by other infectious agents such as bacteria, fungi, and parasites is minimized with advancements in care protocols and biosecurity at tissue banks [14]. Hence, corneal xenotransplantation may be safer than other organs in terms of xenozoonosis development.

With the establishment of regulatory bodies [26] and the initiation of studies in human patients [78], some of these issues are likely to be clarified in the near future.

## Disclosure

The authors have nothing to report.

## Conflicts of Interest

The authors declare no conflicts of interest.
